#  Development of a Simple RP-HPLC-UV Method for Determination of Azithromycin in Bulk and Pharmaceutical Dosage forms as an Alternative to the USP Method 

**Published:** 2013

**Authors:** Tayebeh Ghari, Farzad Kobarfard, Seyed Alireza Mortazavi

**Affiliations:** a *Department of Pharmaceutics, School of Pharmacy, Shahid Beheshti University of Medical Sciences, Tehran, Iran.*; b *Department of Pharmaceutical Chemistry, School of Pharmacy, Shahid Beheshti University of Medical Sciences, Tehran, Iran.*; c *Students Research Committee, School of Pharmacy, Shahid Beheshti University of Medical Sciences, Tehran, Iran. *

**Keywords:** Azithromycin, HPLC, UV detection, Determination, Pharmaceuticals

## Abstract

The present study was designed to develop a simple, validated liquid chromatographic method for the analysis of azithromycin in bulk and pharmaceutical dosage forms using ultraviolet detector. The best stationary phase was determined as C18 column, 5 μm, 250 mm × 4.6 mm. Mobile phase was optimized to obtain a fast and selective separation of the drug. Flow rate was 1.5 mL/min, Wavelength was set at 210 nm and the volume of each injection was 500 μL. An isocratic methanol/buffer mobile phase at the ratio of 90:10 v/v gave the best separation and resolution. The proposed method was accurate, precise, sensitive, and linear over a wide range of concentration of azithromycin. The developed method has the advantage of using UV detector compared to the USP method in which electrochemical detector has been used. The validated method was successfully applied to the determination of azithromycin in bulk and pharmaceutical dosage forms.

## Introduction

**Figure 1:**
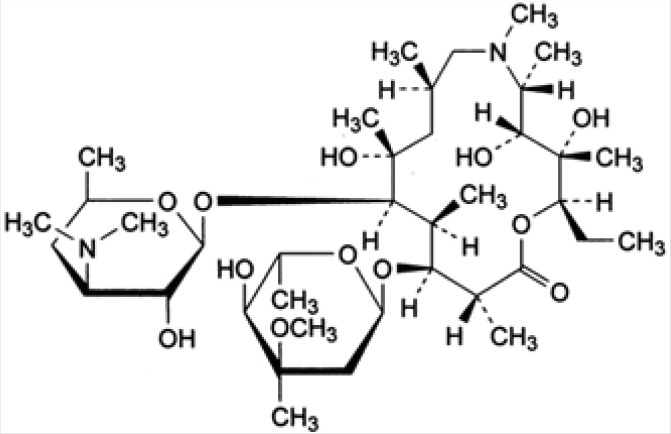
Structure of azithromycin

Azithromycin (AZI) is a semi synthetic macrolide antibiotic with a 15-membered azalactone ring. It is derived from erythromycin; however, it differs chemically from erythromycin in that a methyl-substituted nitrogen atom is incorporated into the lactone ring (as shown in Figure 1). 

Like erythromycin, it appears to bind to the same receptor, 50 S ribosomal subunits of susceptible bacteria and suppresses protein synthesis. It is used primarily to treat various bacterial infections, such as aerobic gram-positive microorganisms and aerobic gram-negative microorganisms. The incorporation of the nitrogen into the ring significantly alters the chemical, microbiologic and pharmacokinetic properties of AZI. It exhibits a more extensive spectrum of activity, greater acid stability and more favorable pharmacokinetic parameters than erythromycin (1). 

Several methods have been developed for determination of AZI in pharmaceutical dosage forms. These methods include high-performance liquid chromatography (HPLC) and microbiological methods. Chromatographic separation is one of the essential and powerful components of the most quantitative analyses and HPLC is currently the most versatile tool which satisfies the needs for an optimum separation (2). AZI has been analyzed by high-performance liquid chromatography using fluorescence (3-5), electrochemical (using amperometric and coulometric detectors) (6-7) and mass spectrometry detector (8-11) for quantification in bulk material and pharmaceutical dosage forms. Fluorescence detection requires complicated sample pretreatment involving pre-column derivatization of the analyte. Assay procedures making use of electrochemical detection is often very time consuming, both in the sample preparation steps and the chromatography. The USP method (12) describes a high pH mobile phase (pH 11) as well as a specific column (Gamma alumina) which is quite expensive and difficult to obtain commercially as many of the column manufacturers do not supply this column. Also, the USP method employs amperometric electrochemical detection, which is not available in many laboratories. Mass spectrometry methods may have the highest sensitivity, but the determination process is complex (13).

A spectrophotometric method has been reported by Suhagia et al for determination of AZI in pharmaceutical dosage forms in which AZI is oxidized with potassium permanganate to liberate formaldehyde, which is then determined in situ using acetyl acetone, in the presence of ammonium acetate. Although the method is simple and easy to perform, it suffers from the lack of separation in determination process which makes it unsuitable for pharmaceutical formulation with unknown excipients that might interfere with UV absorption reading for AZI (14). 

Mallah et al have used FT-IR transmission spectroscopy for determination of AZI in its pharmaceutical dosage forms which is easy to execute but has the same short come of not being suitable for formulations with unknown composition of excipients (15).

Therefore, there is a need for a convenient and effective method for determination of AZI in pharmaceutical dosage forms. Liquid chromatography with UV detection has been already employed for the analysis of AZI in tablets (16) and in raw material (17). In the current work, an HPLC method with UV detector was developed for the determination of a lower concentration of AZI in pharmaceutical dosage forms. Different reversed stationary phases have been compared with each other and their performance has been determined. The final method was selective and sensitive which enables the determination of AZI at 1 μg/mL concentration with good accuracy using UV detector.

## Experimental

*Materials*

AZI standard (99.49%) was supplied by Shifa PharMed Industrial Group (Iran) as gift sample. HPLC-grade methanol, HPLC-grade acetonitrile, potassium dihydrogen phosphate, hydrochloric acid and sodium hydroxide were from Merck (Darmstadt, Germany). HPLC grade water used in the analysis was prepared by reverse osmosis and passed through a 0.45 μm millipore filter (Millipore Company, USA) before use. A pH 6.0 phosphate buffer (0.2 M), was prepared by dissolving 6.8 g of of potassium dihydrogen phosphate in 500 mL of water, adding 28 mL 0.2 M sodium hydroxide solution in water and diluting with water to 1000 mL (USP). Phosphate buffer pH 8.0 was prepared by dissolving 6.8 g of potassium dihydrogen phosphate in 500 mL water, adding 230.5 mL 0.2 M sodium hydroxide solution in water and diluting with water to 1000 mL (USP).

*Equipment*

*Instruments*

HPLC system (D-7000, Merck Hitachi, Tokyo, Japan) with an L-7100 Binary pump, L-7420 UV-Vis detector with column oven (waters, USA) was employed. HSM-7000 software was used for data acquisition and processing. 

*Analytical columns*

AZI was analyzed by reversed-phase HPLC analysis using different HPLC columns. MZ-Analysentechnik GmbH, Perfectsil target, ODS-3 (250 mm length, 4.6 mm inner diameter and 5 μm particle size) column with MZ C18 analytical guard column (20 mm × 4.6 mm) packed with the same sorbent, Lichrospher RP-8, Merck, (250 mm length, 4 mm inner diameter and 5 μm particle size), Lichrospher RP-8, Merck, (100 mm length, 4.6 mm inner diameter and 5 μm particle size), Lichrospher RP-18, Merck (250 mm length, 4.6 mm inner diameter and 10 μm particle size), ODS-H optimal, Capital, (150 mm length, 4.6 mm inner diameter and 3 μm particle size) and C_18_-SB-ZX_3_, Zorbax (50 mm length, 2.1 mm inner diameter and 3.5 μm particle size) were used. All experiments were employed in the isocratic mode.

*Determination of appropriate UV wavelength*

A suitable wavelength was required for determination of AZI. The appropriate wavelength for the detection of the drug in mobile phase was determined by wavelength scanning over the range of 200–400 nm with a Shimadzu® UV-1201 (Shimadzu, Japan) spectrophotometer.

*Chromatographic conditions*

Two organic solvents (acetonitrile and methanol), different volume fractions of a filtered and degassed methanol and acetonitril (50, 60, 70, 80, and 90 v/v) and phosphate buffer with different pH (6.0, 8.0) at concentrations of 0.2 M, 0.02 M were examined as possible mobile phases. Wavelength was set at 210 nm. This wavelength was selected because it is a UV maximum and provides the sensitivity needed for quantitation of the low drug concentration in pharmaceutical dosage forms. The column temperature was maintained at Different temperatures (25 °C, 50 °C). The mobile phase was pumped at different flow rates (1.0, 1.5 mL/min) with 500 μL injection volume.

*Preparation of Standard solutions*

An accurately weighed quantity of AZI (10 mg) was transferred to a 10 mL volumetric flask, approximately 5 mL of the mobile phase was added and dissolved. The solution was brought to volume by the mobile phase and properly mixed to obtain a final concentration of 1.0 mg/mL. The prepared stock solution was stored at 4°C in a glass vial. From this stock solution, standard solutions were freshly prepared prior to analysis.

*HPLC method validation*

Method validation was performed on the best determined stationary phase *i.e*. C18 column, 5 μm, 250 mm × 4.6 mm. 

*Linearity*

The calibration curves were constructed with nine concentrations (simultaneously prepared) ranging from 1 to 80 μg/mL for AZI. Each concentration level was prepared in triplicate and analyzed three times. Calibration curves were constructed by plotting the concentration of compounds versus peak area response. The linearity was evaluated by the least square regression method. 

*Precision*

The precision of the method was determined by repeatability (intra-day) and intermediate precision (inter-day) and was expressed as RSD (%). Nine replicate injections of the standard solutions of AZI at concentrations ranging from 1 to 80 μg/mL prepared as described above. The intra-day variation was assessed by the same analyst over one day, while inter-day precision was carried out by another independent analyst over 3 days (18).

*Accuracy*

The accuracy of the method was tested by replicate analysis of different samples of AZI at known concentrations and then compared with the true concentration of it. Accuracy was assessed by the recovery percentage (18).

*Detection and quantitation limits (sensitivity)*

Limit of quantification (LOQ) was determined during the evaluation of the linear range of calibration curve. LOQ was defined as the lowest concentration yielding a precision (%CV) and accuracy (% recovery ) within their acceptable range with a peak area of three times the limits of detection (LOD) (18).

## Results and discussion

*Method development*

*Wavelength selection*

**Figure 2:**
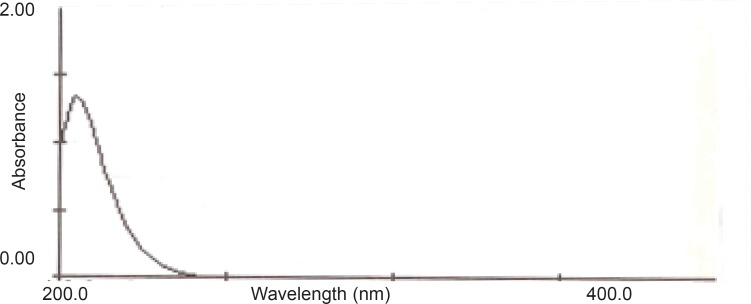
UV spectrum of AZI

The ultraviolet spectra of AZI showed the maximum absorption wavelength at 210 nm (as shown in Figure 2). Therefore, 210 nm wavelength was selected to achieve the highest sensitivity for the study.

*Selection of mobile phase*

**Figure 3:**
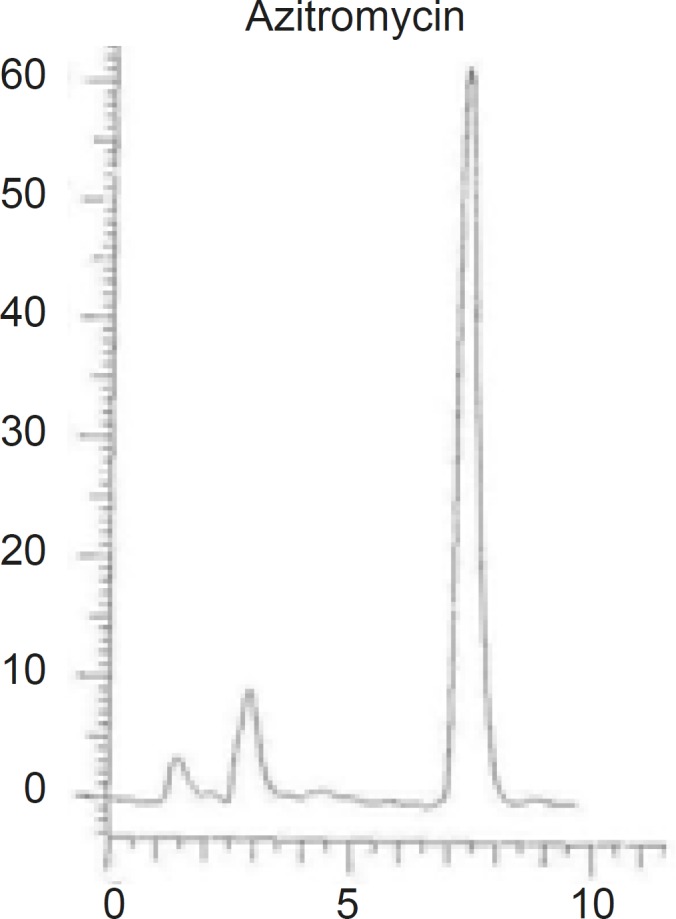
Chromatograms of AZI using MZ-Analysentechnik GmbH, Perfectsil target, ODS-3 (250 mm length, 4.6 mm inner diameter and 5 μm particle size) column

Different combinations of acetonitril or methanol and phosphate buffer, as well as different buffer concentrations (0.2 M, 0.02 M ) were tested and the optimum condition at methanol-phosphate buffer 0.02 M (90:10, v/v), was reached. The obtained chromatogram showed a rapid separation with retention time of AZI at 7.23 min (Figure 3).

*Selection of HPLC stationary phase*

Different reversed stationary phases were used and the results were compared. The best results were obtained by using octadecylsilane (ODS, 18). Various C18 columns with different length and particles size were tried and the results were compared based on the peak width and peak symmetry. The best peak width was obtained on a C18 column with 250 mm length and 5 μm particles size (as shown in Figure 3). 

**Figure 4:**
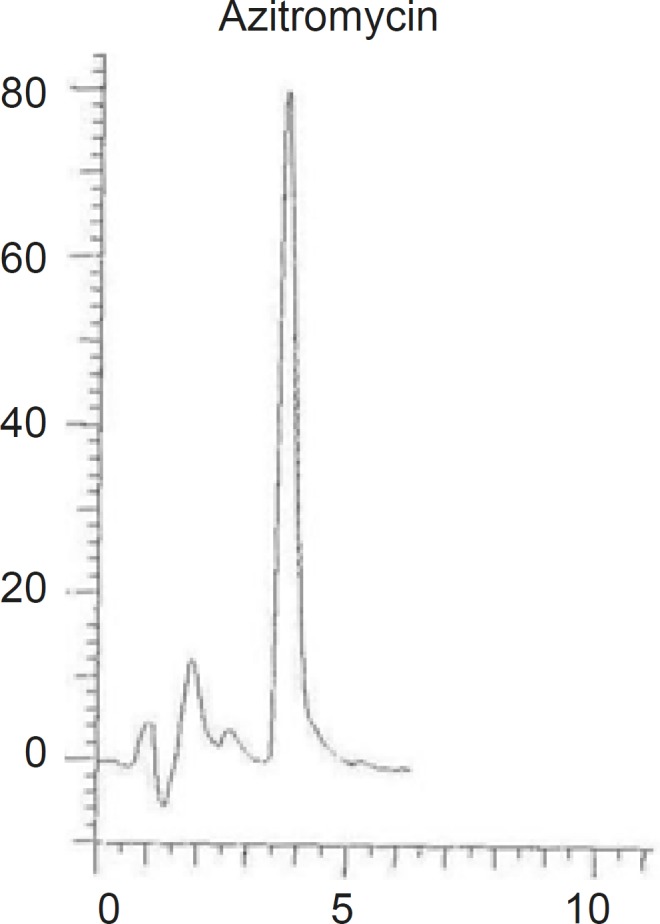
Chromatograms of AZI using ODS-H optimal, Capital, (150 mm length, 4.6 mm inner diameter and 3 μm particle size) column.

Decreasing the length of the column to 150 mm although resulted in shorter retention time, but the peak symmetry was deteriorated. Changing the particle size of the C18 stationary phase from 5 μm to 3 μm resulted in tailing and loss of peak symmetry (Figure 4).

**Figure 5:**
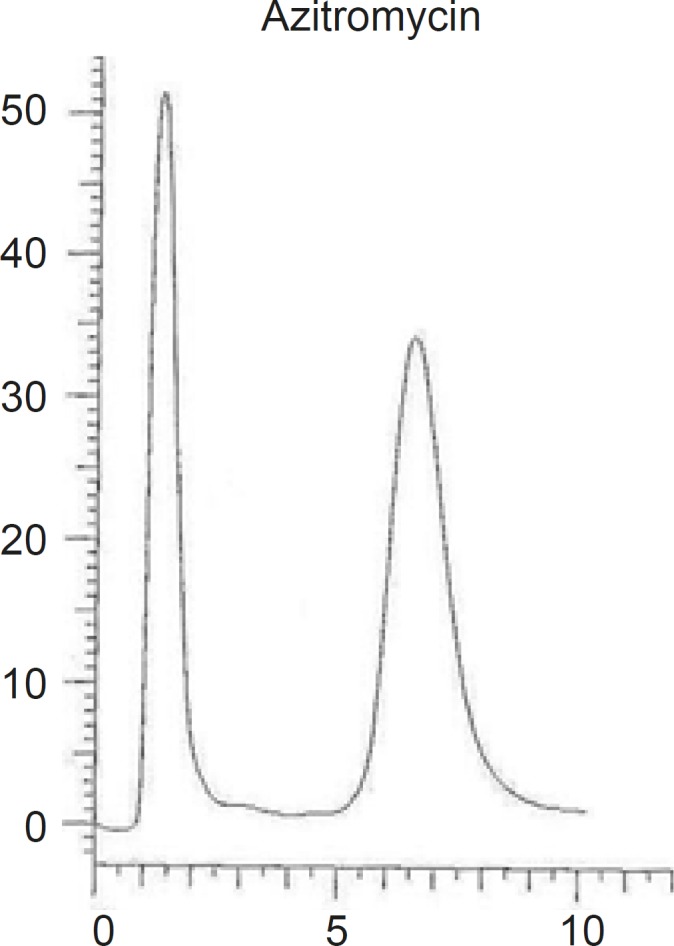
Chromatograms of AZI using Lichrospher RP-18, Merck (250 mm length, 4.6 mm inner diameter and 10 μm particle size) column

Increasing the particle size to 10 μm although gave perfectly symmetric peaks, but increased peak width up to 5 min with lower reproducibility and higher detection limit (as shown in Figure 5).

*Selection of flow rate and column temperature*

Increasing the column temperature from 25 μ to 50 μ led to a decrease in the total time required for the separation process with decrease of peak broadening and increase in sensitivity. Also, increasing the flow rate from 1 mL/min to 1.5 mL/min showed a similar decrease in the retention time. 

*Effect of mobile phase pH*

We studied the effect of varying the pH (6, 8), using 10 % sodium hydroxide solution. Phosphate buffer with high pH (8) was used to avoid problems with silica dissolution. Moreover, the stability of AZI is low in acidic media. We observed that the best separation results were achieved at pH 8.

*Validation of the method *

*Linearity*

**Figure 6:**
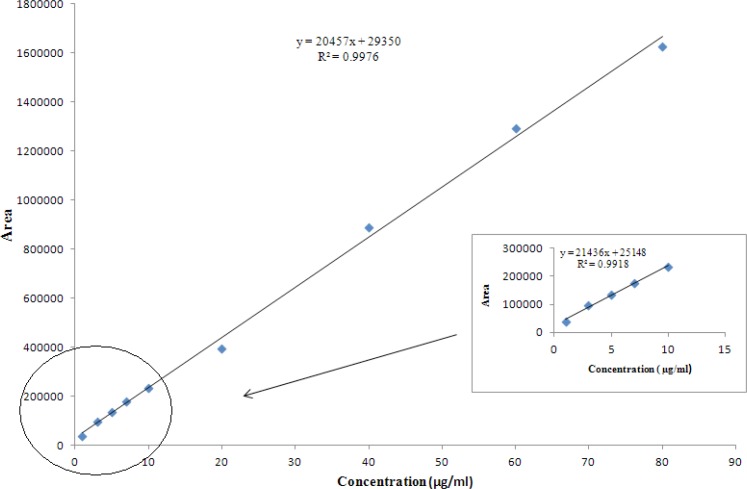
Linearity plot for AZI drug substance

The plot of peak area responses against concentration of AZI is shown in Figure 6. The plot is linear over the concentration range of 1-80 μg/mL yielding a regression equation *Y*= 2.05 ×10^4^*X *+ 2.94×10^4 ^with a coefficient of correlation of 0.9976 and with confidence intervals at p = 0.05. A similar plot at low concentrations (1-10 μg/mL) gave a slope value of 2.14 ×10^4^ (see the inserted box in Figure 6). 

*Precision*

The results obtained for repeatability studies and for intermediate precision are presented in Table 1. Method precision has a relative standard deviation (RSD) below 1.59% for repeatability and 1.61% for intermediate precision, which comply with the acceptance criteria proposed (RSD: not more 2.0%) (19). 

**Table 1:** Intra and inter-day variations of the HPLC method for determination of AZI

**Concentration (μg/mL) **	**Intra-day precision ( % RSD) **	**Inter-day precision (% RSD) **
1	1.29	1.49
3	1.2	1.18
5	0.75	1.27
7	0.98	1.08
10	1.59	0.75
20	1.09	0.88
40	1.79	1.08
60	0.79	1.16
80	1.49	1.61

*Accuracy*

The results were expressed as percent recoveries obtained for different AZI concentration. Table 2 shows that the percent recoveries ranging from 85 to 115.4% with RSDs. ranging from 0.59 to 1.59 % which comply with the acceptance criteria proposed (% Recovery range : 80- 120%) (19). 

**Table 2:** Accuracy/recovery of the proposed method

**Concentration(μg/mL) **	**% Recovery **	**%R.S.D. **
1.5	110.2	1.19
4	115.4	1.59
6	97.9	1.09
8	90.09	0.79
15	85.39	0.89
30	105.4	1.29
50	101.2	0.69
70	85	0.59

*Detection and quantitation limits (sensitivity)*

Results showed that the detection and quantitation limits for AZI using this method are 0.3 μg/mL and 1 μg/mL, respectively. 

## Conclusion

A new, specific, and validated method for the analysis of AZI by using HPLC equipped with UV detection at 210 nm was developed. This method is accurate, precise, sensitive, and linear. This method can be employed for the analysis of AZI at various concentrations and in different drug formulations as well as raw material. 
